# Naturally Occurring Variability in the Regulatory Region of the *Drosophila melanogaster* Gene *shuttle craft* Affects Gene Expression and Lifespan

**DOI:** 10.3390/ijms27188343

**Published:** 2026-09-19

**Authors:** Alexander V. Symonenko, Natalia V. Roshina, Olga Y. Rybina, Evgeniya A. Tsybul’ko, Anna V. Krementsova, Elena A. Mikhaleva, Vladimir E. Alatortsev, Dmitry V. Mukha, Elena G. Pasyukova

**Affiliations:** 1 National Research Centre “Kurchatov Institute”, 2 Kurchatov Sq., 123182 Moscow, Russia; roschina_nv@nrcki.ru (N.V.R.); flybee@mail.ru (O.Y.R.); jen@cos.ru (E.A.T.); krementsova@sky.chph.ras.ru (A.V.K.); pasyukova_eg@nrcki.ru (E.G.P.); 2 Vavilov Institute of General Genetics, Russian Academy of Sciences, 3 Gubkin Str., 119991 Moscow, Russia; dmitryvmukha@gmail.com; 3 Emanuel Institute of Biochemical Physics, Russian Academy of Sciences, 4 Kosygin St., 119334 Moscow, Russia

**Keywords:** lifespan, transcription, transcription factors, natural populations, polymorphism, SNP, cold tolerance, *Drosophila melanogaster*

## Abstract

Revealing gene expression patterns is essential for comprehending the genetic control of complex traits like lifespan. Analyzing structural variation in gene regulatory regions within natural populations is a promising approach in functional genetics, allowing us to identify regulatory sites that influence transcription and phenotype in vivo. We describe the structural variability of the 5′ regulatory region of the *shuttle craft* (*stc*) gene in the Alexandrov population (Russia) of *Drosophila melanogaster*, encoding a transcription factor homologous to human NF-X1. We evaluated the association of frequently occurring polymorphisms with fly lifespan from inbred lines sampled in 2010, 2011, and 2014. Two polymorphisms in the *stc* 5′ regulatory region exhibit strong associations with both lifespan and embryonic *stc* transcript levels. Their role in expression regulation was evaluated via reporter gene assays conducted in S2 *Drosophila* cell culture. The ‘C’ allele at one polymorphic site and ‘T’ at the other are associated with increased longevity and decreased embryonic *stc* transcription, reaffirming the link between gene expression and lifespan we previously established. Furthermore, comparing *stc* 5′ regulatory region variability across Alexandrov, European and African populations suggests that the macro-geographic distribution of these longevity-associated variants is consistent with a potential role in latitudinal or climatic adaptation.

## 1. Introduction

In multicellular organisms, precise regulation of both transcriptional and posttranscriptional programs is crucial for maintaining the accurate relationship among genomic information, individual cell identities, functional attributes of tissues and organs, and the resulting phenotype of the entire organism. The coordination of multiple distant enhancers, extensive alternative promoter usage, and numerous splicing events allows for exact and sophisticated regulation of gene expression. The complexity of the transcriptome and cis-regulatory DNA sequences, which control transcript abundance and diversification, mirrors the complexity of a living being [1]. Simultaneously, in higher organisms, the regulatory machinery exhibits evolutionary conservation and functional similarity, making *Drosophila melanogaster* (*D. melanogaster*) an effective model for functional genomics studies.

Unraveling the gene expression strategy is essential for comprehending the genetic control of complex traits, such as lifespan. The significance of such studies increases when considering biologically and socially significant quantitative traits, unequivocally including longevity. In recent years, the human lifespan has been on the rise, highlighting the value of life extension, particularly when linked with decelerated aging and the preservation of health. In this context, the search for effective molecular and cellular mechanisms to extend healthy longevity, including the potential to influence gene expression levels, has become an increasingly pressing task. In our previous work, we demonstrated that lowered embryonic transcription of the *shuttle craft* (*stc*) gene, which encodes a transcription factor involved in the control of neuronal development, led to increased longevity [2]. Furthermore, reduction in the *stc* transcript levels in embryos through RNA interference also resulted in increased lifespan [3]. Therefore, in laboratory experiments, the regulation of *stc* expression levels underlies changes in lifespan, suggesting the potential for controlling this important trait at the transcriptional level. Nevertheless, the question remains open as to how well the effects of experimental interventions reflect the patterns that exist in natural conditions.

The presence of substantial genetic and phenotypic variation in nature is widely acknowledged. Extensive data have facilitated the assessment of demography, ecological adaptation, and selection in natural populations (for example, [4,5,6,7,8,9]). Unraveling the genetic basis of natural trait variation is one of the most challenging objectives in population genetics, aiming to elucidate the connections between variations in gene structure and specific phenotypes. Significant progress has been achieved over the last few decades in comprehending the relationships between variations in gene structure and various phenotypic traits of the fruit fly, including lifespan, as well as genetic variation at both the single-gene and whole-genome levels (see for examples [10,11,12,13,14,15,16,17]).

The effects of natural genetic variation on lifespan and other life history traits in *D. melanogaster* were for example recently reviewed by T. Flatt [18]. Much less is known about the effect of genome variation, including its regulatory sequences, on gene transcription and furthermore, about the relationship of this altered transcription with phenotypic changes. Recently, the integration of deep DNA sequencing with RNA sequencing or metabolome profiling of inbred lines derived from natural populations of *D. melanogaster* has brought us closer to bridging the gap between natural genomic variation and quantitative traits. This advancement stems from the analysis of changes in so-called intermediate phenotypes, namely, transcriptomes and metabolomes [19,20,21,22,23]. In the years to come, the increasing flow of publications describing natural variation in gene expression and its associated phenotypes will improve our understanding of the genetic architecture of populations and illuminate the evolutionary forces that sustain both intra- and inter-populational diversity.

On a more precise, single-locus scale, it has been demonstrated that natural variation in the regulatory regions of *D. melanogaster* genes, including *blw*, *CG9509*, and *MtnA*, is associated with variations in gene expression and affects quantitative traits, such as lifespan [24], oxidative stress tolerance [25,26,27], adult body size, and wing loading [28], respectively. We also demonstrated that two naturally occurring polymorphisms located in the *Lim3* 5′ regulatory region were associated with a six-fold change in gene transcription in embryos and a 25% change in lifespan. Through a reporter gene approach, we obtained experimental evidence confirming that *Lim3* transcript amounts in embryos depended on the presence of specific nucleotides at each of the two polymorphic sites located in the putative binding sites of several regulatory proteins within the active Polycomb Response Element. Importantly, markers associated with long life and intermediate *Lim3* transcription were present in the population at high frequencies [29,30]. We believe that this line of research is not only crucial for describing the molecular basis of population dynamics and biodiversity but also for advancing our understanding of functional genomics and the regulation of gene expression in general.

In this paper, we explore structural variation in the 5′ regulatory region and 5′ UTR of the *stc* gene in the Russian population of *D. melanogaster*, specifically from the Alexandrov subset. Subsequently, we compare these findings with data from several European and African populations, as described by others (http://dpgp.org, [8,31,32]). Based on this comparison, we selected frequently occurring polymorphic markers characteristic of all populations and assessed the association of these markers with the lifespan of inbred lines obtained from individual females captured in the Russian population of Alexandrov in 2010, 2011 and 2014. Our findings reveal that two polymorphic markers located in the *stc* 5′ regulatory region are associated with both lifespan and *stc* transcript amounts in embryos. Their capacity to modulate transcription was demonstrated via reporter gene assays conducted in S2 *Drosophila* cell culture. Specifically, the presence of ‘C’ in one of the polymorphic sites and ‘T’ in the other appeared to be associated with an increase in lifespan and a decrease in *stc* transcription in embryos. These results further confirm our previous findings, establishing a clear link between gene expression and the nature of its impact on longevity. A comparison of our findings with published data also suggests that the geographical distribution of these longevity-associated variants is consistent with a potential role in climate adaptation.

## 2. Results

Our research focused on monitoring natural variation in *D. melanogaster* from the Alexandrov population located in the northern region of European Russia (Table A1). Flies were collected from late August to early October, a period when they were present in substantial numbers.

To evaluate the naturally occurring variation in DNA sequences and lifespan, we employed a well-known approach (for example, [33,34]) based on the analysis of ILs obtained from individual females captured in the wild through full-sib matings. According to [35], every IL is, to a reasonable extent, genetically homozygous and formally represents one haploid genome (one allele) from a natural population. In practice, however, ILs may retain heterozygous fragments. While this situation can present challenges for whole-genome sequencing, it becomes less problematic when sequencing small regions of the genome because if ILs exhibit residual heterozygosity in the region of interest, they can be selectively discarded. Considering both the limitations and benefits of this approach, which allows for the analysis of not only DNA polymorphism but also the naturally occurring variation in quantitative traits, we selected from 18 to 43 ILs per population per year for subsequent study (Table A1).

**Figure 1:**
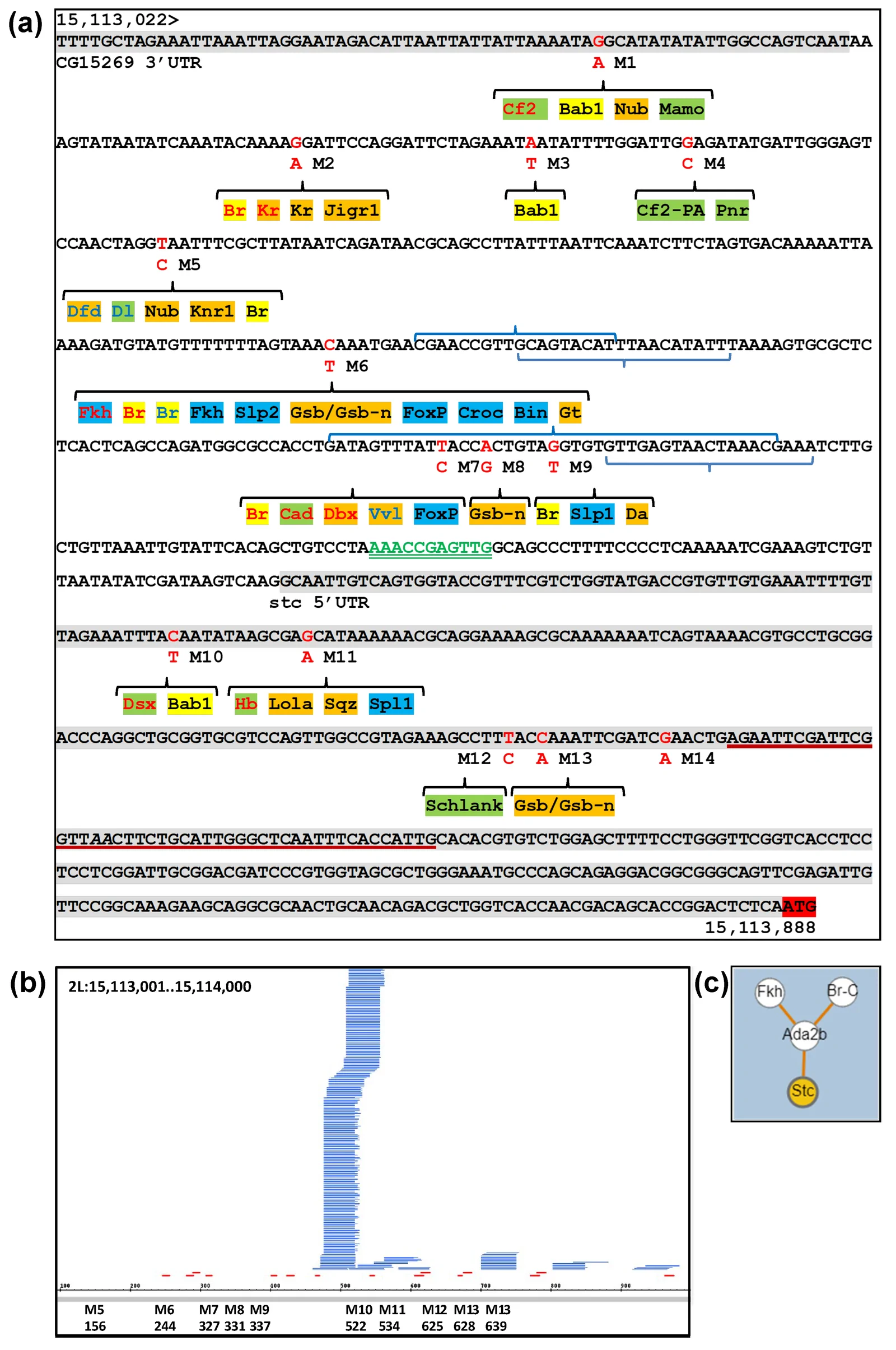
Structure of the *stc* 5′ regulatory region. (**a**) The sequence of the regulatory region and the locations of the polymorphic markers. Polymorphic markers (M1–M14) are highlighted in bold red letters. The 3′ UTR of the *CG15269* gene and the 5′ UTR of the *stc* gene are shaded in light grey, with the *stc* translation start site marked in red. Transcription factors predicted to bind to polymorphic sites, as identified by JASPAR, LASAGNA and Tomtom (see Section 4 for details), are denoted in red, blue, and black letters, respectively, and are highlighted in different colors: yellow for BTB family transcription factors; orange for neuronal transcription factors; blue for Forkhead family transcription factors; and green for other transcription factors. The insulator is indicated in green letters and underlined with a double line, while regulatory RNA-binding sites are underlined with a thick red line. The full names of the genes/proteins can be found at http://flybase.org. (**b**) The positions of core promoter elements and the region of dispersed transcription start sites. The 5′-ends of mRNAs (red) and possible elements of the *stc* gene promoter (blue) are found using FIMO (https://meme-suite.org/meme/doc/fimo.html (accessed on 20 August 2026)) based on [37] as described in Section 4. (**c**) Possible Stc interactions with factors with predicted regulatory motifs at M5 and M6 (visualized by esyN after http://flybase.org).

To determine whether *stc* expression and lifespan are associated with the naturally occurring structural variation in the putative cis-regulatory region of *stc*, we sequenced 868 bp from 109 ILs obtained from the Alexandrov population, including 71 bp of the 3′ UTR of the preceding gene, *CG15269*, 386 bp of DNA sequences located upstream of *stc*, and 409 bp of the *stc* 5′ UTR (Table A1; Figure 1). *CG15269* and *stc* are closely linked, and previous studies (namely, [36]) have shown that the insertion causing the *stc[05441]* mutation, exhibiting the lethal phenotype typical of classic *stc* mutations, is actually located within an adjacent gene, *CG15269*. The relationship between these two genes remains unclear, as does the function of *CG15269* (https://flybase.org/reports/FBgn0028878 (accessed on 20 August 2026)). We included the 3′ end of *CG15269* in our analysis in order to increase the size of the DNA fragment preceding the structural part of *stc*, suggesting that it may also be part of the *stc* 5′ regulatory region. In the next section, we present a brief analysis of molecular variation in the cis-regulatory region of *stc*, using only fully read homozygous DNA sequences without any omissions.

### 2.1. DNA Polymorphism in the cis-Regulatory Region of the shuttle craft Gene

**Figure 2:**
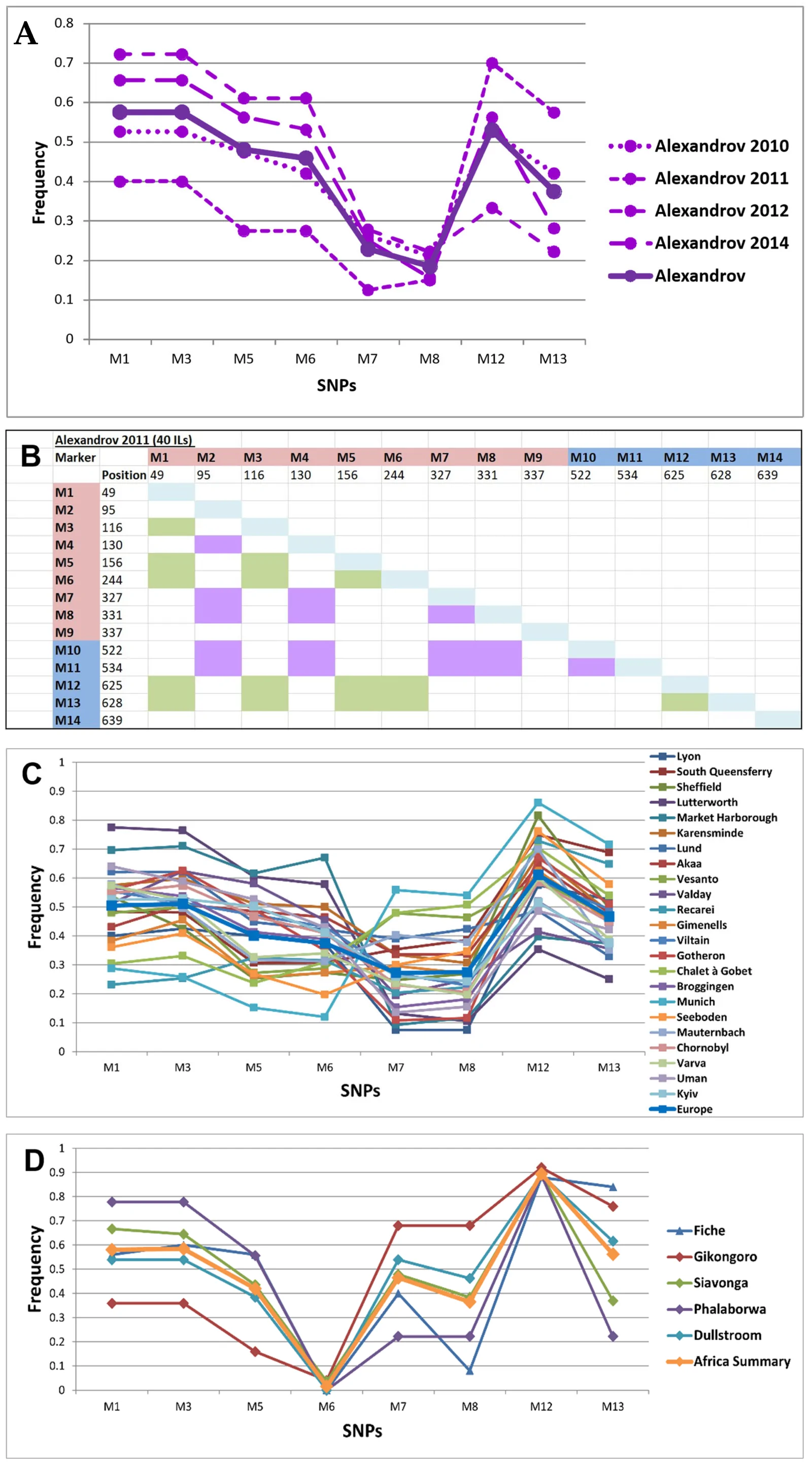

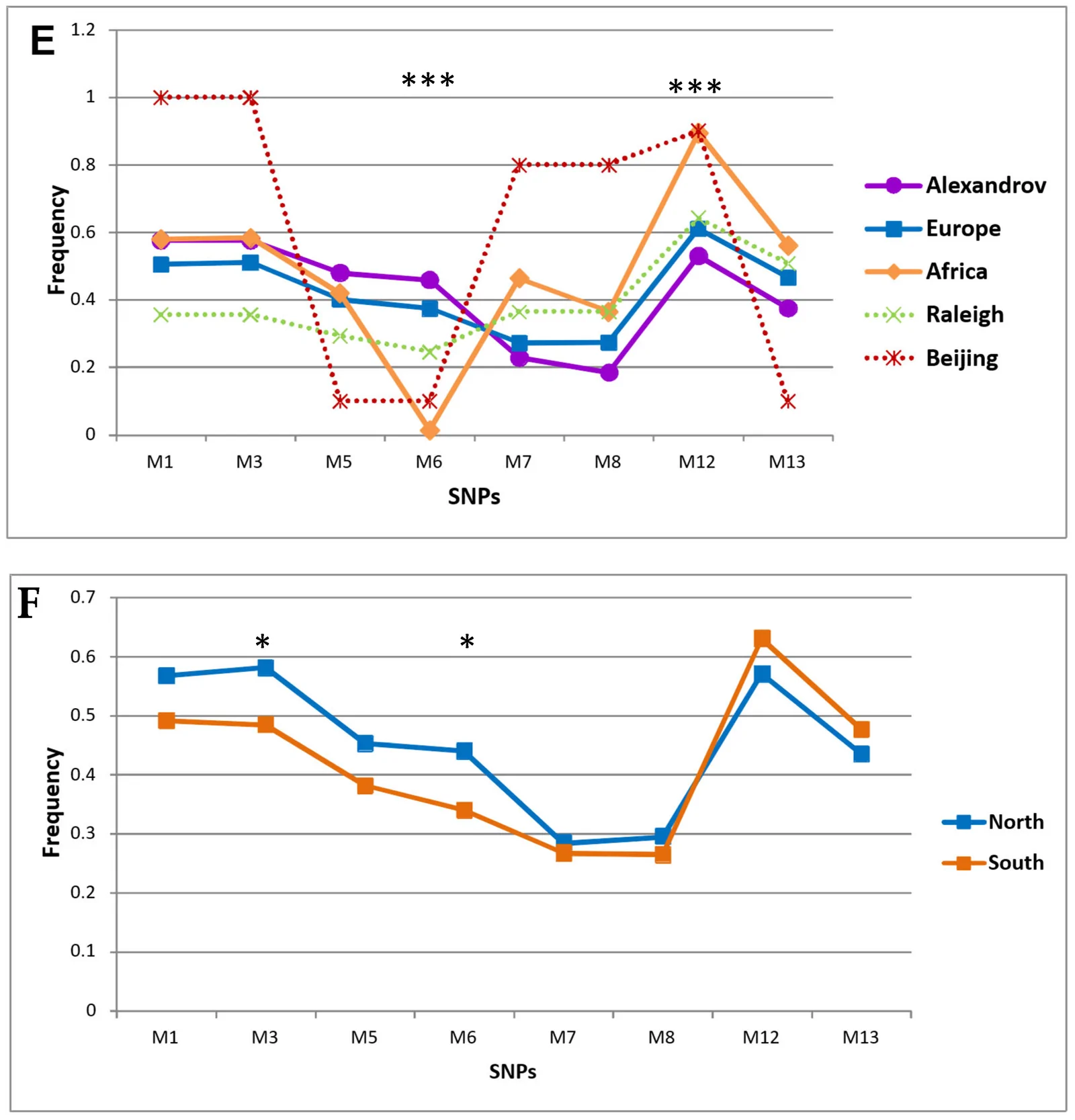
Natural variation in the *stc* 5′ regulatory region. (**A**) Marker frequencies in the Alexandrov population in different years; (**B**) LD in the Alexandrov population in 2011. Different colors represent nominal linkage groups maintained for consistency across figures and tables, with dark teal explicitly highlighting the linkage group containing markers most significantly associated with adult lifespan. Exact LD numerical values are detailed in Appendix A Table A1; (**C**) Marker frequencies in European populations, utilizing data from [8]; (**D**) Marker frequencies in African populations, utilizing data from [31,32]; (**E**) Averaged marker frequencies in Alexandrov, European, and African populations. Frequencies for the Raleigh and Beijing populations [31,32] are included for comparison. M5 was represented by three nucleotides in African populations, and was consequently removed from the graph. ***—*p* < 0.001, ANOVA; (**F**) Marker frequencies in northern and southern European populations, utilizing data from [8]. *—*p* < 0.05, ANOVA.

The number of nucleotide substitutions found in the Alexandrov population varied from 15 to 19 in different years. Among these, 14 SNPs occurred with a frequency equal to or greater than 5% and will be further referred to as polymorphic markers, M1–M14 (Supplementary Materials, List S1). Estimates of nucleotide diversity, based on the number of differences between pairs of sites (π, [38]), and the number of segregating sites (θ, [39]), fell within the observed range for *D. melanogaster* [40]. Specifically, π ranged from 0.00518 ± 0.00056 to 0.00604 ± 0.00064 (Table A1), and θ ranged from 0.00486 ± 0.00118 to 0.00626 ± 0.00144. No significant difference in nucleotide diversity was found between the years, and each year, the *stc* 5′ UTR was slightly more conserved than the *stc* 5′ regulatory region (Table A1). Comparing the frequencies of the eight most common SNPs truly segregating in the natural population (frequency greater than 10%) by year (Supplementary Materials, List S2; Figure 2A) revealed substantial changes: 2011 stood out as the most different year from the rest, whereas 2012 and 2014 showed the most similarity. The frequencies of SNPs averaged across years almost entirely corresponded to the frequencies observed in 2010. The cause of the year-to-year changes in marker frequencies remains unclear. Regarding weather conditions, summer 2010 was exceptionally hot in European Russia (https://world-weather.ru/archive/russia/aleksandrov/summer/ (accessed on 20 August 2026)), while other years experienced normal weather conditions. It is hardly reasonable to assume that special marker frequencies in 2011 were predetermined by exceptional weather in summer 2010, since the flies were caught in autumn, and there are no grounds to believe that the survival of the population during the cold winter period of 2010–2011 should have affected the frequencies differently than in other years. The frequencies of different markers changed in concert, indicating a fairly strong linkage between SNPs. Six of the eight frequent markers (M1, M3, M5, M6, M12, M13) formed a specific group of significantly linked polymorphic sites (Supplementary Materials, List S3; Figure 2B illustrates this for 2011). In the same year, an additional linkage pattern was also observed for M2, M4, M7, M8, M10, M11 (Supplementary Materials, List S3; Figure 2B). Overall, the observed patterns of linked loci deviated from the expectations of normal recombination.

To understand whether the pattern of molecular variation identified in the Alexandrov population was similar to patterns in other populations, we compared Alexandrov with other populations for which full-genome DNA sequences determined in 10 or more ILs obtained from populations at the same time (2010–2014) were available (DPGP2 and DPGP3 collections, http://dpgp.org, [31,32]): one European (Lyon, France) and five African (Fiche, Ethiopia; Gikongoro, Rwanda; Siavonga, Zambia; Phalaborwa and Dullstroom, South Africa). Only fully read DNA sequences without any omissions in the region of interest were used. Parameters of nucleotide diversity were similar in Alexandrov and all other populations (Table A1). Similar to Alexandrov, in African populations, the *stc* 5′ UTR was slightly more conserved than the *stc* 5′ regulatory region, whereas in the French population, the difference was not evident (Table A1). Similar to the Alexandrov population, in the French population, M1, M3, M5, M6, M12, and M13 formed a distinct group of significantly linked polymorphic sites (Supplementary Materials, List S3). Conversely, this pattern was not observed in the African populations; instead, in the Gikongoro and Siavonga populations M1, M3, M4, M7, and M8 exhibited linkage disequilibrium (LD, Supplementary Materials, List S3).

Molecular genetic tests for selection were used to determine whether evolutionary forces might be regulating nucleotide variation in the *stc* 5′ regulatory region. Notably, no significant values for D [41] (Table A1), D* and F* [42] were found in any of the populations.

The same polymorphic markers (M1–M14) were identified in all populations (refer to Supplementary Materials, List S1). In the Alexandrov and Lyon populations, only these SNPs were present. However, in African populations, nine additional SNPs with a frequency equal to or greater than 5% were found (A1–A9, Supplementary Materials, Lists S1 and S2). In recent years, a large amount of data on the variability of the *D. melanogaster* genome in natural populations has been obtained by pool sequencing [43]. To better assess the variability of the *D. melanogaster* genome in European natural populations, we used data obtained by the European *Drosophila* Population Genomics Consortium (DrosEU; https://droseu.net, [8]). In addition to the M1–M14 polymorphic markers found in all 22 European populations in 2014, we identified 26 SNPs with frequencies equal to or greater than 5% in one (16 SNPs), two (3 SNPs), three (3 SNPs), five (2 SNPs), six (1 SNP), or seven (1 SNP) populations (E1–E26, Supplementary Materials, List S2). Notably, only one of these additional sites matched an extra site in the African populations (A4 = E8).

The frequencies of the M1–M14 polymorphic markers varied within both European and African populations (Figure 2C,D). Interestingly, the average yearly frequencies of M1–M14 in the Alexandrov population closely resembled the average European frequencies (Figure 2E). Against the background of the general variability in the frequencies of polymorphic markers in individual populations, two exceptions were noted: the frequencies of M6 and M12 were nearly identical in all African populations. The average frequencies of these same markers were found to be significantly different in European and African populations (Figure 2E). This finding implies a potential association between polymorphisms at these sites and adaptation to the fly environment. Indeed, all populations inhabit a relatively narrow band between 3 degrees West and 33 degrees East. Notably, European populations reside significantly farther North than African populations (latitude range of 58 to 41 degrees North for Europe compared to 10 degrees North to 25 degrees South for Africa). Thus, we hypothesize that the low frequency of T in M6 and the high frequency of C in M12 are characteristic of southern populations. To verify this assumption, we compared the frequencies of M6 and M12 in northern and southern European populations, dividing them conventionally into two groups of approximately equal numbers based on a geographic latitude threshold of 50° N (where populations > 50° N were categorized as northern and populations < 50° N as southern; Supplementary Materials, List S2). The frequencies of M6 and M12 in these groups indeed differed in accordance with expectations, providing a pattern consistent with a potential role in climatic or latitudinal adaptation (Figure 2F).

In conclusion, variation in the *stc* 5′ regulatory region differs between European and African populations, in accordance with similar population genetic observations [44,45]. Notably, the variation in the Alexandrov population aligns with that of other European populations and, to some extent, in the African population, which allowed the use of it as a representative population for investigating the effect of nucleotide diversity in the *stc* 5′ regulatory region on gene expression and fly lifespan.

### 2.2. Exploring the Association Between Molecular Variation in the stc 5′ Regulatory Region and Lifespan

**Figure 3:**
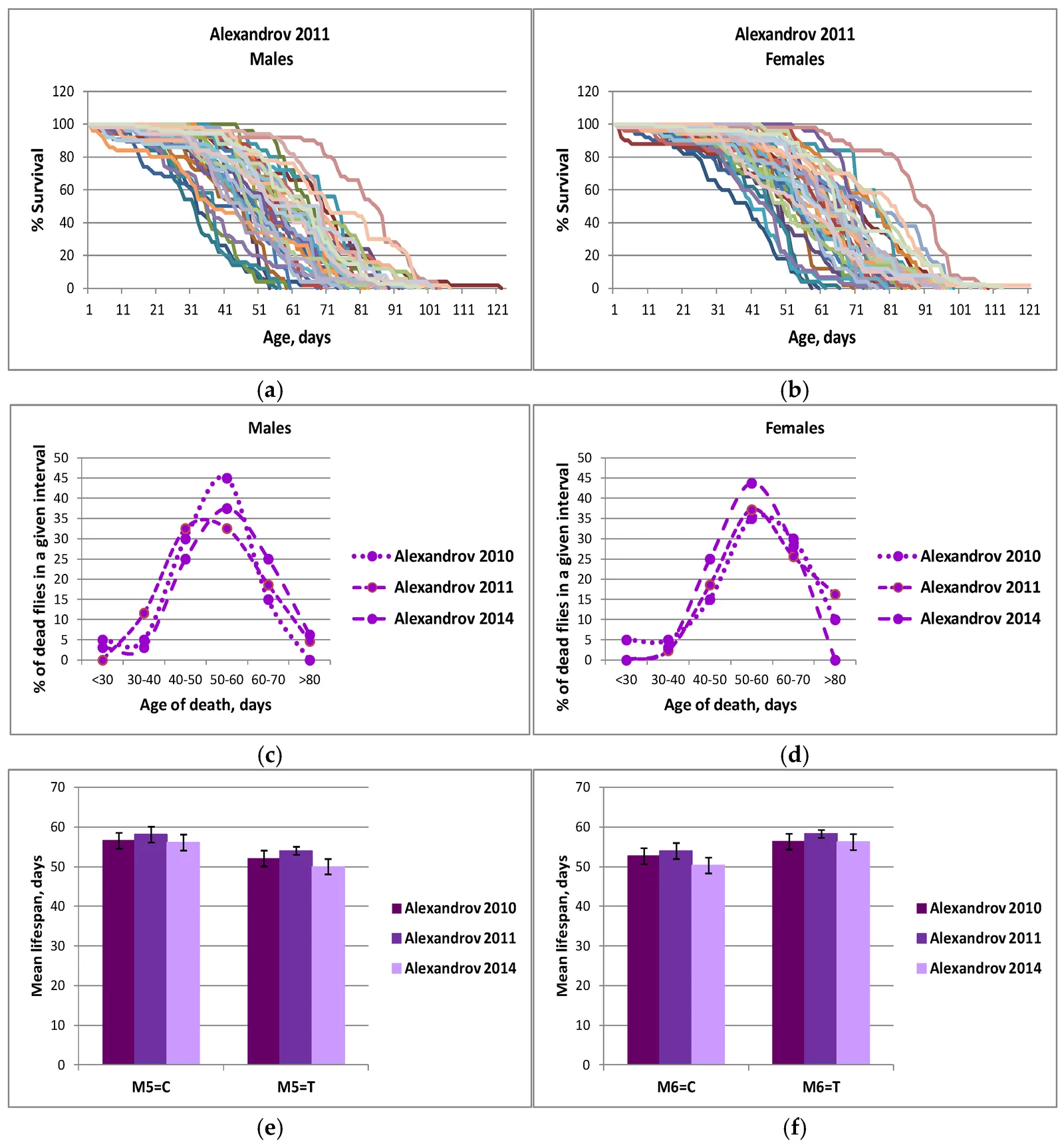
Lifespan in ILs with different naturally occurring polymorphisms. (**a**,**b**) Survival curves for males and females of ILs obtained from the Alexandrov population in 2011 (individual lines are colored arbitrarily for visual differentiation); (**c**,**d**) Frequencies of dead flies from ILs obtained from the Aleksandrov population at different time intervals; (**e**,**f**) The mean lifespans of flies from ILs with C and T alleles in markers M5 and M6.

To assess associations between the naturally occurring structural variation in the cis-regulatory region of the *stc* gene, known to be involved in lifespan control [2], and lifespan, we measured lifespan in ILs derived from the Alexandrov 2010, Alexandrov 2011, and Alexandrov 2014 populations (Supplementary Materials, List S4). This included four lines with missing data for one or two markers, which were not included in the analysis of nucleotide diversity. We observed substantial variation in survival between the lines in both males and females (Figure 3a,b). At the same time, variation between the years was not essential (Figure 3c,d).

Association studies utilized 8 polymorphic markers present in our samples at a frequency of 10% and higher. This restriction, also employed in the analysis of essential characteristics of the structural variation in the *stc* 5′ regulatory region, allowed us to focus on polymorphisms that were genuinely segregating in nature.

Analysis of variance (ANOVA) did not reveal any significant association with lifespan for sex, year, or any interaction term (Supplementary Materials, List S5). Based on these results, we combined data on sexes and years for the analysis of associations between polymorphic markers and lifespan. Four markers (M1, M3, M5, and M6) showed a significant association with lifespan (Table 1, Supplementary Materials, List S5). These markers, located in the *stc* 5′ regulatory region (Figure 1), were in significant LD with each other (Supplementary Materials, List S3). However, ANOVA revealed that the association between the haplotype composed of the four markers and lifespan was much less significant than the association between any individual marker and lifespan (Table 1). Nonparametric, distribution-free Kruskal–Wallis tests were used to evaluate the significance of the difference in lifespan between the ILs with different nucleotides in M1, M3, M5, and M6 sites, as well as with different haplotypes, and generally confirmed the results obtained from ANOVA (Table 1).

Although M12 and M13, located in the *stc* 5′ UTR, were also in significant LD with M1, M3, M5, and M6 (Supplementary Materials, List S3), no association with lifespan was observed for M12 and M13 (Supplementary Materials, List S5).

Two major combinations of markers, GATC and ATCT, composed of M1, M3, M5, and M6, were present in the Alexandrov population (Table 1). A multiple comparison of means showed that only the lifespans of ILs with these haplotypes were significantly different. The mean lifespan of ILs with the minor combination of markers, ATTC, the only other combination that truly segregated in the population and thus had a reliable estimate of lifespan, did not formally differ from the lifespan of ILs with both major combinations of markers. However, it was closer to the mean lifespan of ILs with the GATC combination (Table 1). This suggested that the contribution of variation in markers M1 and M3 to the variation in lifespan was less significant than the contribution of markers M5 and M6, which were most strongly associated with lifespan. Indeed, only the significant *p*-values for the association between the marker allele and lifespan at M5 and M6 survived the Bonferroni correction for multiple tests. It is relevant to recall that the M1 marker is actually located in a gene other than *stc*, and M1 and M3 are closely linked to M5 and M6, which may explain their association with longevity. The mean lifespans of flies from ILs with different alleles in markers M5 and M6 remained quite constant over time (Figure 3e,f).

We hypothesized that polymorphisms in the regulatory region of the *stc* gene affect a phenotypic trait, such as lifespan, because they influence gene expression. Our next goal was to test this hypothesis.

### 2.3. Exploring the Association Between Molecular Variation in the stc 5′ Regulatory Region and stc Expression

Four annotated *stc* transcripts, two of them experimentally demonstrated to produce proteins (RA and RB differing by 21 nucleotides due to alternative splicing [36]) are reported (https://flybase.org/reports/FBgn0001978 (accessed on 20 August 2026)). *stc* is predominantly expressed in embryos. According to modENCODE Temporal Expression Data (https://flybase.org/reports/FBgn0001978 (accessed on 20 August 2026)), the level of *stc* mRNA is rather steady throughout embryogenesis, with very high amounts observed at early stages, and high amounts observed up to 18 h of development. Higher amounts of *stc* mRNA in early embryos may be explained by the presence of maternally derived transcripts [46]. In larvae, pupae, and adults, *stc* transcripts are present at low to moderate levels in most tissues, with the exception of high amounts of *stc* mRNA observed in ovaries. Previously, we demonstrated a substantial decrease in the level of *stc* transcription in mutant *stc*^KG01230^ embryos but not in larvae or adults of any age, whether virgin or mated. This result, coupled with the fact that *stc*^KG01230^ caused an increase in lifespan, led us to hypothesize that the regulation of *stc* expression during early development could be a determining factor for adult lifespan [2].

**Figure 4:**
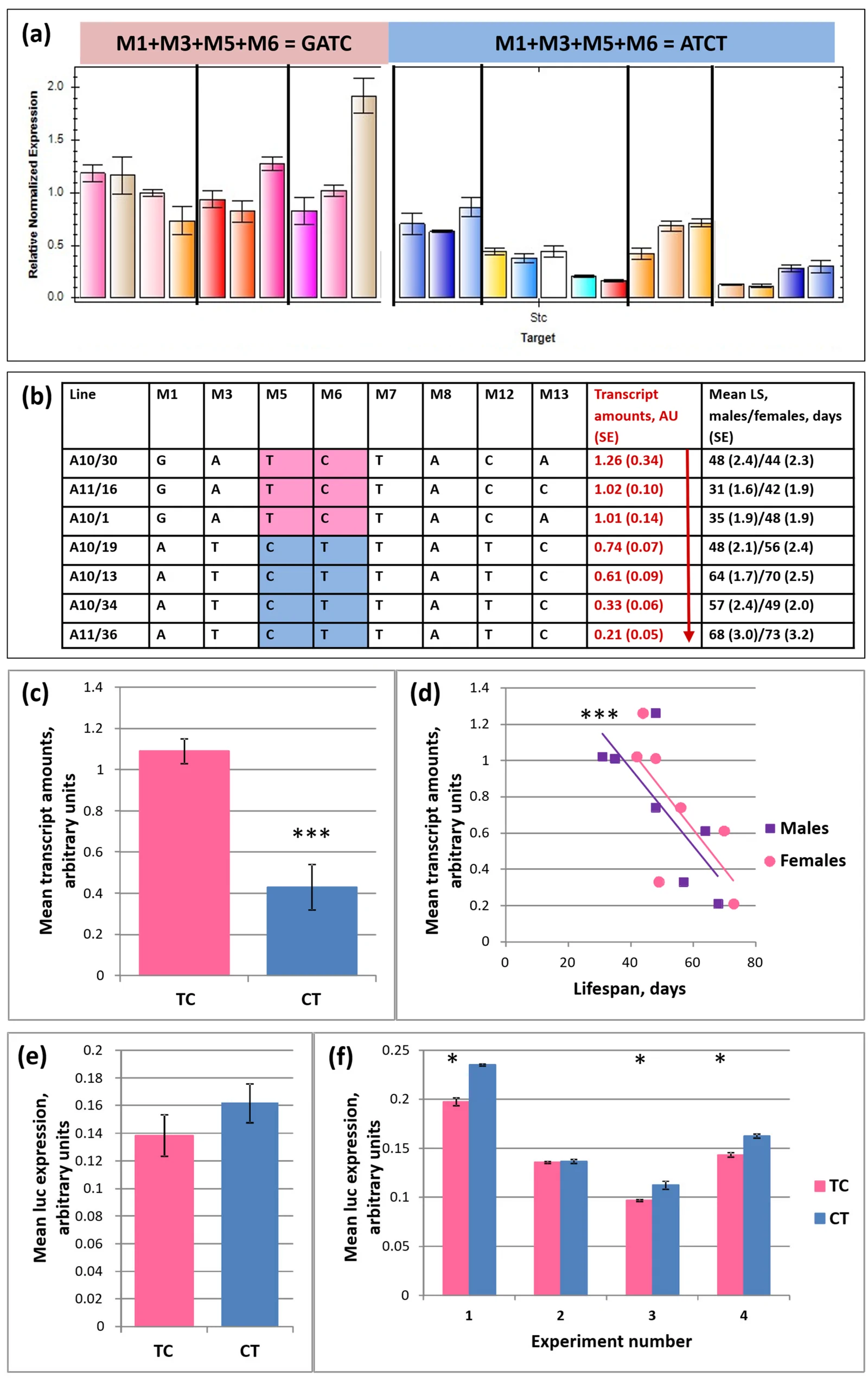
*stc* transcript amounts in ILs with different naturally occurring polymorphisms. (**a**) *stc* transcript amounts determined by RT qPCR in selected ILs obtained from the Alexandrov population. Individual bars represent technical replicates for each specific IL, arranged in a sequential descending order that corresponds directly to the data matrix in (**b**); (**b**) polymorphic markers, *stc* transcript amounts, and lifespan in selected ILs obtained from the Alexandrov population. Pink and blue highlighted markers are significantly associated with lifespan. The red arrow indicates a decrease in the *stc* transcript amounts; (**c**) mean *stc* transcript amounts in ILs with different haplotypes in the *stc* 5′ regulatory region. True biological sample size is determined by the number of independent inbred lines (*N* = 3 for TC lines, *N* = 4 for CT lines). ***—*p* < 0.001, Kruskal–Wallis test; (**d**) correlation between *stc* transcript amounts and lifespan in selected ILs obtained from the Alexandrov population. (Pearson’s r = −0.7839, R^2^ = 0.6145, *p* = 0.0213). ***—*p* < 0.001; (**e**,**f**) luciferase expression controlled by CT or TC combinations of markers M5 and M6 averaged over four experiments and shown per experiment, respectively. Error bars represent the Standard Error of the Mean (SEM). *—*p* < 0.05, Kruskal–Wallis test.

Guided by this information, to assess the impact of naturally occurring variation in the *stc* 5′ regulatory region on *stc* transcription, we evaluated the amounts of *stc* transcripts in 12–24 h embryos using three ILs with TC and four ILs with CT combinations of markers M5 and M6 (Figure 4a,b). Nucleotides at the other six SNPs that were present in our samples at a frequency of 10% or higher are shown in Figure 4b.

Since it is impossible to directly measure the sole RA transcript amount, we estimated the total *stc* transcript amount. ANOVA revealed a highly significant association between the marker combination and *stc* expression (*p* < 0.0001), whereas the specific association of lifespan with the marker combination was borderline non-significant (*p* = 0.0511). The Kruskal–Wallis test confirmed that *stc* transcript amounts were significantly different in ILs with the TC and CT genotypes (*p* < 0.0001; Figure 4c). The presence of C in M5 and T in M6 appeared to be associated with both increased lifespan (Figure 3e,f) and decreased *stc* expression (Figure 4c). Indeed, a highly significant correlation between the mean lifespans and *stc* expression was observed in males, females, and when both sexes were combined (*p* = 0.0269, *p* = 0.0234, and *p* = 0.0003, respectively; Figure 4d).

To directly assess the functionality of the naturally occurring polymorphisms and experimentally evaluate the regulatory potential of DNA sequences located upstream of the structural part of the *stc* gene, we performed a reporter gene assay in *D. melanogaster* cell culture.

### 2.4. Effects of Molecular Variation in the stc 5′ Regulatory Region on the Reporter Gene Expression in the S2 Cell Culture

To generate reporter constructs incorporating the firefly luciferase gene, regulated by the *stc* 5′ regulatory region, the relevant sequences with CT or TC combinations of markers M5 and M6 were inserted into the pGL3-Basic vector (Promega, Madison, WI, USA). Schneider S2 cells were transfected with one or the other of the two pGL3 vectors containing different structural variants of the *stc* 5′ regulatory region, or with the control pGL3-Basic vector. Relative luciferase activity was measured in transfected cells. In all experiments, luciferase expression in control samples was three orders of magnitude lower than in other cases.

According to ANOVA, the effect of the CT/TC combination on the level of reporter gene expression was highly significant (*p* = 0.0042). At the same time, ANOVA showed a significant effect of the replicate experiment (*p* = 0.0001), which led to significant errors in the mean expression values. Therefore, it was not surprising that the reporter gene expression, controlled by CT or TC combinations of markers M5 and M6, was not significantly different when averaged across all experiments (*t*-test: *p* = 0.1681; Kruskal–Wallis test: *p* = 0.3118; Figure 4e). Importantly, in all four replicate experiments, the direction of differences in reporter gene expression controlled by CT or TC combinations remained consistent (in ANOVA, the interaction term—replicate experiment by marker combination—was not significant), and in three experiments the difference in reporter gene expression was significant (*t*-test: *p* = 0.006, *p* = 0.0011, *p* = 0.0008; Kruskal–Wallis test: *p* = 0.0495 for each comparison; Figure 4f). Thus, we concluded that the presence of nucleotides T or C in the M5 and M6 positions can influence *stc* expression. Notably, the direction of the effect in the cell culture experiments turned out to be opposite to the direction of the effect in the ILs (Figure 4).

### 2.5. Structure of the stc 5′ Regulatory Region

To further confirm the potential functional significance of the identified polymorphic sequences, we studied their correlation with evolutionarily conserved sites using the phastCons and phastConsElements prediction tracks in the UCSC Genome Browser. We compared the level of conservation by analyzing a broader and narrower spectrum of insects (127 species vs. 24 species, including *Drosophilidae* and a few others), considering that the difference in conservation levels detected by these two approaches would reveal relatively more or less long-standing changes. Of the 14 polymorphisms found in Alexandrov, M6, M7, M8, M10, and M11 appeared to be located in the conserved areas of the *stc* regulatory region revealed for both the group of 127 and 24 insect species. However, the conservation scores for M6, M7, and M8 were somewhat unexpectedly smaller for the last group (Supplementary Materials List S2). This paradox can possibly be explained by the fact that, although located within conserved areas, these polymorphisms (M6, in particular) are subject to directional selection. This is further supported by their nucleotides having higher selection scores compared to the surrounding nucleotides, as determined based on phyloP data. Apparently, these polymorphisms are located in adaptively important sites within essential and, therefore, conserved regulatory structures in the regulatory region of the gene. This suggestion is in perfect agreement with the significance of these polymorphic sites for determining the expression level of *stc* demonstrated in this work.

The CAGE data for S2 cells (SRA ID: SRR6508014, [47]) were used to map the 5′-end of the *stc* transcripts. The mapping revealed that the *stc* gene exhibits dispersed transcription initiation, with multiple transcription start sites (TSSs) spread over a 66 bp region. Notably, both M10 and M11 fall within the TSS area. Core promoter motifs [37] in the *stc* 5′ regulatory region were determined using FIMO [48] with a significance threshold of *p* < 10^−4^ (Figure 1b). The major promoter motifs 7 and 2 [37] are located in the regions between nucleotides 424 and 436, and nucleotides 465 and 472, respectively, directly upstream of the TSS (Figure 1b). Motif 2 corresponds to the DNA replication element sequence, which is highly conserved among the core promoters of several *Drosophila* genes. Importantly, no polymorphisms were detected in this crucial region.

It is reasonable to hypothesize that naturally occurring sequence variations in the gene 5′ regulatory region could change binding sites of regulatory proteins and RNAs, contributing to differences in gene expression levels. To elucidate the potential transcription factors binding to the regulatory region of the *stc* gene, we first analyzed data from the modENCODE project (modENCODE Consortium et al., 2010 [49]). The information from this project is arguably more relevant as it describes experimentally identified transcription factors present in this region. However, the project data are limited to the stages of development at which the analysis was performed, and the accuracy of determining the boundaries of transcription factor binding sites (TFBSs) does not exceed hundreds of nucleotides. According to the modENCODE analysis, the embryonic transcription factor binding hotspot, TFBS_HSA_004897, spans the entire intergenic region between *CG15269* and *stc*. It is associated with seven transcription factors, including those involved in the control of nervous system development during late embryogenesis, namely, *H*, *Disco*, *Da*, *D*, *Dl*, *Twi*, and *Cad*. Additionally, in white prepupae, enrichment of the Br transcription factor in the region was reported. Our analysis of the raw data for embryos revealed a very similar Broad enrichment peak (MACS_peak_790, 2L:15,113,012..15,113,899).

Using several databases, we predicted TFBSs in the regulatory region of the *stc* gene. Since predictions from a single algorithm or application are usually less reliable, we have prioritized the TFBSs simultaneously predicted by different approaches. We used the JASPAR 2022 prediction for the *Drosophila* genome along with the JASPAR 2022 CORE insects collection, the LASAGNA 2.0 application with the Transfac Public database, and Tomtom v.5.5.0 software with Combined *Drosophila* Databases, all with their default settings, to find the major transcription factors likely to be located in the *stc* regulatory region (Figure 1a; Supplementary Materials, List S6). LASAGNA and JASPAR predict TFBSs in the entire selected region of the genome, while Tomtom predicts TFBSs within a specified frame of a certain size (11nt or 21nt) around the SNPs in question. This combination of approaches allows us to consider the most likely motifs within the entire area and provide a reasonably accurate prediction of motifs associated specifically with the SNP in question.

Among the transcription factors potentially regulating *stc* transcription are key proteins involved in the control of early embryogenesis (http://flybase.org; *Dsx*, *Hb*, *Cad*, and others), as well as those affecting the development of the nervous system (http://flybase.org; *Kr*, *Dl*, *Gt*, *Pnr*, *Br*, *Nub*, *Da*, *Slp1*, *Slp2*, *FoxP*, *Gsb*, and others). In total, 14 transcription factors related to the development and function of the nervous system, which can potentially bind to the *stc* regulatory region, were revealed (Figure 1a; Supplementary Materials, List S6). Both JASPAR 2022 and Tomtom predicted *Kr* TFBS at M2 and *Slp1* TFBS at M11. However, the JASPAR 2022 prediction score for *Slp1* was slightly below the threshold level (Supplementary Materials, List S6). Similarly, at M6, both Tomtom and JASPAR2022 predicted TFBSs for *Slp1* and *Slp2,* although the JASPAR2022 score was slightly below the threshold level (Supplementary Materials, List S6). Given their known similar expression patterns, particularly in the embryonic head [50], and their tendency to form functional complexes during neuronal cell specification [51], the simultaneous prediction of *Slp1* and *Slp2* in the same area makes their cooperative presence appear more likely. Also, at M6, a TFBS for the Broad transcription factor was predicted by both JASPAR 2022 and LASAGNA. The JASPAR 2020 prediction specifically associates the predicted motif matching M6 with Br (var.4), suggested to be involved in the development of certain groups of neurons [52]. Several TFBSs are predicted for transcription factors belonging to the Fork head box family: *Fkh*, *Croc*, *Bin*, *Slp1*, *Slp2*, and *FoxP*. Forkhead TFBS at M6 is predicted by both JASPAR 2022 and Tomtom. The fact that the DNA sequences surrounding M6 are enriched with predicted binding sites of various transcription factors (Figure 1a) reinforces the assumption that this site may be involved in the regulation of transcription. Of all the transcription factors for which binding sites in the *stc* regulatory region were predicted, only *Cad*, *Dl*, and *Br* were found there experimentally, according to the modENCODE project data [49].

The 11-nucleotide insulator was detected in the *stc* 5′ regulatory region (https://flybase.org/jbrowse/?data=data%2Fjson%2Fdmel&loc=2L%3A15110854..15122423&tracks=Gene_span&highlight= (accessed on 20 August 2026)). Interestingly, the putative binding site of the CTCF, a ubiquitous transcription factor that binds to insulators and domain boundaries, was found nearby (Figure 1a). Also, three overlapping target sites for regulatory RNAs (Dme-mir-1010, Dme-mir-1012, Dme-mir-303) were found in the *stc* 5′ UTR (http://www.flymine.org/query/ (accessed on 20 August 2026)). However, no polymorphic sites were detected within these sequences (Figure 1a).

Overall, this brief analysis showed that the *stc* 5′regulatory region has good regulatory potential, and nucleotide substitutions revealed in populations might well affect gene transcription and, as a result, a complex phenotype and lifespan. Notably, the M6 marker associated with *stc* transcription and lifespan is located in the DNA region most rich in potential TFBSs.

## 3. Discussion

In this paper, we demonstrated that naturally occurring polymorphism in two sites located in the 5′ regulatory region of the *stc* gene was associated with variation in a complex quantitative trait, lifespan, and the level of *stc* transcription. It remains to be elucidated whether these results are characteristic of this particular population or if they will be reproduced in other populations, and if yes, whether it would depend on their habitat or not. Work in this direction is currently in progress, but we already have reason to believe that the trends identified are universal, since both population studies and laboratory experiments demonstrated that it is the reduction in the *stc* transcription level at the embryonic stage that leads to an increase in longevity. We believe that analysis of the natural variation in gene regulatory regions followed by laboratory tests has several advantages for studying molecular mechanisms controlling gene expression compared to purely laboratory studies. The population approach allows us to find precisely those fragments of the regulatory region of a gene that effectively influence transcription and phenotype in vivo and to assess the subtle effects of changes in individual nucleotides on these traits using both genome-wide [19] and individual gene [30] analyses.

To prove the causative role of a particular naturally occurring variation in the control of gene expression and phenotype and to exclude hitchhiking effects, in several recent publications, the study of genotype-transcription associations in natural populations has been supplemented by direct experimental verification of the effects identified. For example, it has been demonstrated that three closely linked SNPs located in the regulatory sequences controlling the expression of the pigmentation gene tan and associated with pigmentation variation in natural *D. melanogaster* populations [53] affected abdominal and thoracic pigmentation in transgenic females expressing tan under the regulation of the cloned sequences containing candidate SNPs [54,55]. The impact of SNPs on gene expression has been confirmed by analyzing their effect on reporter gene expression in cell cultures and different tissues of transgenic flies. Five of the six cloned genetically variable upstream regulatory regions of P450 genes demonstrated different gene expression patterns in the renal tissues of transgenic flies [20]. We have explored in detail the role of variation in the regulatory region of the *Lim3* gene in controlling reporter gene expression in embryos and various larval and adult tissues [30]. The effects of three SNPs and various parts of the core and distal promoter, including putative binding sites of regulatory proteins, were first evaluated in transfected cell cultures and then confirmed in transgenic flies.

Here, we evaluated the effects of two SNPs located in the *stc* 5′ regulatory region on gene expression. While the effect of single substitutions in the *Lim3* regulatory region on gene expression in cell culture has not been detected [30], the effect of SNPs located in the *stc* 5′ regulatory region, although small, was significant. The modest impact on reporter gene expression was anticipated, because it was caused by changing just two nucleotides in the DNA sequence. The direction of the effect in experiments with cell culture was opposite to the direction of the effect initially observed in ILs. This directional discrepancy, combined with a non-significant pooled average across the biological replicates, highlights a clear limitation of our in vitro assay. Thus, the reporter construct should be interpreted as evidence of regulatory capacity rather than a direct confirmation of endogenous in vivo expression mechanics. Even though the expression was measured in embryos and in the S2 cells derived from embryos, one obvious explanation for this fact may be attributed to different regulatory signals in living organisms compared to a particular cell culture strain. In particular, the S2 cells are commonly believed to have the characteristics of hemocytes [56], while at least some of the *stc* lifespan effects can be attributed to the changes in its expression in the nervous system [2]. Additionally, given that significant SNPs were in strong LD with each other and several surrounding SNPs in our samples, and could potentially be linked to some distant polymorphisms, it is also possible that the observed discrepancy is due to background effects present in ILs but absent in the reporter constructs. Indeed, background effects have been found to be widespread [11]. Furthermore, it has been demonstrated that most expression variation found in *D. melanogaster* strains from different populations can be attributed to trans-regulation [28]. A plausible hypothesis is that at least several linked markers (M1, M3, M5, and M6) may form a functional haplotype block involved in lifespan regulation and possibly correspond to blocks believed to represent joint targets for selection and adaptation [57]. On the whole, we found our results to be informative and therefore limited the study to cell culture. Despite some inconsistency and regardless of possible background effects, nucleotide substitutions in the M5 and M6 polymorphic sites located in the *stc* 5′ regulatory region were demonstrated to influence gene expression. We believe that further studies should prioritize the analysis of the effects on gene expression of newly identified SNPs in other populations, along with their combinations. This approach would allow us to experimentally address background effects.

### 3.1. Temperature Adaptation

One of the main adaptations associated with moving from south to north is temperature adaptation. Many other factors, such as the influence of environmental humidity and the speed and specificity of animal metabolism, are largely modulated by the ambient temperature, and these factors together pose one of the main challenges for the geographic invasion of species from geographically southern regions to the north. This issue is relevant not only for insects; it is equally important in the long-term adaptation process for higher mammals and humans [58].

Given the observed geographic dependence of the variant frequency distribution of our polymorphism sites, particularly M6, we may speculate that it is also associated with transcription factor motifs regulating *stc* expression, which is also under the pressure of ecological selection. It is known that divergent selection between neighboring thermal environments is often strong enough to maintain a bimodal distribution of genotypes [59].

Since we observe the strongest dependence of our polymorphism site M6 on latitude, with the ‘T’ variant frequency reaching 100% in southern populations, and we assume that this site is part of the predicted binding motif for several Forkhead family TFs (in particular, Fkh and Croc), it is important to understand the possible pathways of their involvement in the overall regulation of temperature adaptation, particularly to cold. Indeed, it is known that the transcriptional regulator FOXC2 of the Forkhead family, homologs of which are known in many mammals, including mice and humans, is involved in cold adaptation regulation [60]. This regulation is believed to be associated with both triglyceride synthesis regulated by the insulin pathway and stimulation of non-shivering thermogenesis through uncoupling of ATP synthesis. Another transcription factor from the Forkhead family, Foxo, is also known to be involved in *Drosophila*’s clinal adaptation through the insulin/insulin-like growth factor signaling pathway, regulating overall fitness [61]. Although Croc is known to have TF-TF interactions with FOXO [62], its known developmental functions lie in head morphogenesis [63], so the greater significance may lie not in the general protein homology but in the specific properties of the Forkhead domain of other TFs, also predicted to bind the motif containing M6, particularly Forkhead, which is known to be involved in more diverse physiological processes, including growth control through the Tor pathway [64], fatty acid metabolism, hormone-regulated signaling pathways [65], and lifespan determination when expressed in adult nerve cells. The same lifespan-controlling effects were reported for glial *foxo* expression [66]. Taking this into account, we may suggest Stc to be another important target of Forkhead lifespan-prolonging regulation in the nervous system.

An alternative mechanism of temperature adaptation in insects is the seasonal regulation of diapause under unfavorable conditions, which is functionally similar to hibernation in other animals. Protein regulators of the Forkhead family may also participate in the regulation of this process [67]. Interestingly, the 5′ regulatory region of *stc* is expected to contain many binding sites for the Br protein, which regulates the response to ecdysone. Its binding sites coincide, including with the M5 SNP, detected near the possible Forkhead-binding M6. Since both proteins have a common partner, Ada2b, similar to Stc, it can be hypothesized that they may mutually regulate each other’s functions and jointly participate in temperature adaptation, alternatively through the regulation of developmental arrest and diapause processes.

Given these possible mechanisms contributing to climatic adaptation, the regulation of *stc* expression, important for latitudinal variability, may be carried out jointly by proteins of the Br and Forkhead families and may be involved in thermogenesis mechanisms (possibly through the regulation of *SERCA* expression being a putative Stc target), as well as through the temporal regulation of organism development processes related to developmental rate, diapause, and developmental arrest.

Studying the role of latitudinal variability in the regulatory region of the *stc* gene (likely associated with cold adaptation), it is also interesting to realize that this evolutionary process likely correlates with changes in lifespan, which, as we have shown, is influenced by *stc* expression. The role of metabolic shifts associated with this adaptation, as well as changes in developmental stage boundaries, is evident, and all these processes may participate in regulating the balance between metabolism rate and aging [68].

Given the potential role of Stc in thermogenesis, another possible function of this protein, in addition to its role in neurogenesis, could be of importance too. It is known that the human homolog of Stc, NF-X1, regulates inflammation [69], and Stc itself may be involved in the Imd pathway signaling through interaction with Tab2 [70]. Thermogenesis and fever are known to be induced in the brain through pro-inflammatory factors [71], so this conservative pathway may also explain the potential role of Stc in regulating thermogenesis.

### 3.2. TFBSs in the stc Regulatory Region

Several potential TFBSs were identified in the vicinity of the M5 and M6 markers. However, not all of these sites may be functional due to the limitations of prediction algorithms and relatively poor ability of eukaryotic transcription factors to specifically recognize target DNA sequences [72]. Given the known functions of endogenous Stc expression during 14–20 h of embryo in neuronal formation and particularly axon guidance [36], it would be reasonable to focus on the motifs for regulatory proteins potentially involved in these processes. The M5 and M6 markers colocalize with the putative binding sites of transcription factors involved in neurogenesis. For example, *Dl* regulates the development of the dorsoventral axis of the embryo, including the laying down of the ventral nerve circuit [73] and development of the peripheral nervous system [74]. Another important transcription factor, *D*, also regulates the development of the dorsoventral axis of the embryo, brain development, and the direction of axon and dendrite growth [75]. *Br* is known to lengthen the *Drosophila* lifespan [76]. Recognition sites for other transcription factors important for nervous system development were also predicted in the *stc* 5′ regulatory region, including *H* (peripheral neurogenesis), *Disco* (brain development), *Da* (neurogenesis) and others (Figure 1). The known functions of Twi, which is potentially capable of binding to the *stc* regulatory region, do not align with those of Stc. Nevertheless, experimental evidence has demonstrated functional interactions between Twist and Daughterless [77,78] and *Twi* and *Dl* [79,80] supporting their involvement in shared regulatory complexes. TFs from the Forkhead box (FOX) family are of special interest. FoxP was predicted to participate in the control of locomotion and various forms of learning and behaviour (http://flybase.org/reports/FBgn0262477 (accessed on 20 August 2026)), while *Fkh* was predicted to impact ageing [81]. TFs regulating other processes also potentially bind to the *stc* regulatory region. Cad, for instance, could be associated with the regulation of another potential function of *stc* related to the control of innate immunity (https://www.kegg.jp/dbget-bin/www_bget?dme:Dmel_CG3647 (accessed on 20 August 2026)).

The colocalization of recognition sites for various TFs within the regulatory region of the *stc* gene indirectly confirms the functional importance of the entire putative TFBSs located in this region. Broad and Forkhead were reported to form complexes with Ada2b, one of the few known protein partners of Stc (according to Flybase data, processed by esyN v.2.1 [82] (Figure 1c). It can be assumed that various factors forming common regulatory protein complexes jointly control Stc participation in transcriptional cascades and nervous system development. Further experiments are required to demonstrate which regulatory proteins indeed bind to the *stc* 5′ regulatory region and whether this binding is dependent on SNPs located in this region. Importantly, the question of how changes in gene expression at the embryonic stage can affect the lifespan of the adult fly remains open. We suggested that the long-term effects of the early-life changes in transcription might be epigenetically inherited in cell lineages. Alternatively, the Stc transcription factor might participate in transcriptional cascades, predetermining structural and therefore functional properties of the adult fly [2]. Testing these assumptions is part of our plans, and the first results certify that, at least at the larval stage, the structure of neurons is altered due to a change in the functional status of the *stc* gene in embryos.

## 4. Materials and Methods

### 4.1. Drosophila melanogaster

*D. melanogaster* females were captured from a private apple garden in a small hamlet, devoid of supermarkets or fruit markets, situated several kilometers from Alexandrov, Russia, at the coordinates (56.41° N, 38.72° E). Flies were manually collected from the surface of apple heaps, spanning late August to early October in the years 2010, 2011, 2012, and 2014.

Each year, we initiated between 40 and 60 isofemale lines from females captured in nature. The phenotype of males in their progeny was examined to prevent possible contamination by *Drosophila simulans*. Although wild-caught *D. simulans* are rare at this locality, caught females were sorted in the lab using the broad pigmentation band on the 5th abdominal segment, and line purity was verified in the F1 generation using male genital traits and diagnostic genetic crosses. Each line underwent 20–22 generations of brother × sister inbreeding, effectively minimizing their genetic heterogeneity. The resulting inbred lines (ILs) were then used for experiments.

### 4.2. Tests for Wolbachia

As detailed earlier [83], prior to the experiments, all ILs underwent qPCR screening for the presence of *Wolbachia*, a symbiont known to affect life history traits in *D. melanogaster* [84]. Detection was performed using the MiniOpticon Real-Time PCR System (Bio-Rad, Hercules, CA, USA) with primers targeting the *Wolbachia* 16S rRNA gene, 5′-CATACCTATTCGAAGGGATAG-3′ and 5′-AGCTTCGAGTGAAACCAATTC-3′ [85]. In the Alexandrov population infection rates for 2010, 2011, 2012, and 2014 were observed at 80%, 82.2%, 67.9%, and 50.7%, respectively [86]. *Wolbachia*-positive lines were treated with tetracycline in laboratory food (0.25 mg/mL [87]) for three generations, followed by three generations of recovery before being utilized in lifespan assays.

### 4.3. PCR and Sequencing

DNA was extracted from batches of 20 flies of each genotype using a standard phenol-chloroform procedure [88]. DNA was used in PCR reactions with primers s15m, 5′-ACCCTTCTCAGCAATCTCAGGA-3′ and pstc2-3m, 5′-CGCCCAGGGAGAAGTTAGTGTA-3′. PCR products were purified with acrylamide gel electrophoresis and sequenced on the ABI PRISM 310 Genetic Analyzer (Applied Biosystems, Waltham, MA, USA) using the sequencing primers s0, 5′-CAAAGTGCAGTACTTCCACTGTT-3′ and as0, 5′-CTGCCATGGCGGATGACTCA-3′ and the Big Dye Terminator V. 3.1. Kit (Applied Biosystems, Waltham, MA, USA), according to the manufacturer’s protocol. DNA sequences were deposited in GenBank.

### 4.4. Lifespan Assay

Lifespan was measured following the protocol detailed in [2]. Five virgin flies of the same genotype and sex, all collected on the same day from cultures with moderate density, were placed in replicate vials. Flies were transferred to vials with fresh food containing approximately 5 mL of standard medium weekly. The number of dead flies was recorded daily. The ILs from Alexandrov in 2010 and 2011 were organized into two groups of 25 lines each. Lifespan measurements were conducted sequentially for each group in turn, with several reference lines included in each experiment. The sample size comprised 25 flies per sex per genotype. Subsequently, the lines were redistributed into groups, and the lifespan measurements were repeated following the same protocol. In two lines the lifespan was measured only once because they died after that. The lifespan measurements for the ILs of Alexandrov 2014 followed the same protocol three years later. The total sample sizes amounted to 50 to 80 flies per line/sex. Lifespan, calculated as the number of days from eclosion to death for each fly, was used to estimate mean lifespans, serving as the primary characterization of longevity in this study.

### 4.5. Real-Time RT-qPCR

As detailed in [83], total RNA for the real-time reverse transcription quantitative PCR (RT-qPCR) was extracted from 50–100 μL of embryos aged 12–24 h. Embryos were collected in PBS buffer on ice, and the extractions were performed using the TRIzol reagent (Invitrogen, Carlsbad, CA, USA) and DNase I (Sigma-Aldrich, St. Louis, MO, USA), following the manufacturers’ instructions.

First-strand cDNA was synthesized from approximately 1 μg of the total RNA using SuperScript II Reverse Transcriptase (Invitrogen, Carlsbad, CA, USA) with oligo(dT)_15_ primers, following the manufacturer’s instructions. The amounts of cDNA were determined by RT-qPCR with SYBR Green I in the MiniOpticon real-time PCR detection system (Bio-Rad, Hercules, CA, USA).

The housekeeping genes *gdh* (glutamate dehydrogenase) and *adh* (alcohol dehydrogenase), characterized by relatively low expression levels comparable to that of *stc*, were used as reference genes to normalize for differences in total cDNA quantity between the samples. The forward and reverse primer sequences used were as follows:

For *stc*:

stc-rt1, 5′-AACAGGCACAGCAACAACAA-3′

stc-rt2, 5′-CCAGGGAGAAGTTAGTGTAGC-3′

For *gdh*:

Gdh1 5′-TATGCCACCGAGCACCAGATTCC-3′

Gdh2 5′-GGATGCCCTTCACCTTCTGCTTCTT-3′

For *adh*:

Adhd3: 5′-CGGCATCTAAGAAGTGATACTCCCAAAA-3′

Adhr3: 5′-TGAGTGTGCATCGAATCAGCCTTATT-3′.

The relative gene expression was evaluated using CFX Manager v. 3.1 software (Bio-Rad, Hercules, CA, USA, 2013). Independent inbred lines (ILs) were treated as the true independent biological units of observation. For each unique IL, three to four independent RNA extractions were performed to serve as biological sub-replicates. Finally, three technical qPCR replicates were executed for each individual RNA extraction. The averaged results of technical replicates were used for further analysis.

### 4.6. Vector Construction for the Dual Luciferase Expression Assay

The 1100 bp fragment located upstream of the structural part of the *stc* gene was PCR-amplified using DNA of the IL originating from the Alexandrov population and the following primers: fw1, 5′-ACCCTTCTCAGCAATCTCAGGA-3′; rev1, 5′-CTGACGTTGCCTCCAGTGCT-3′.

At Evrogen (https://evrogen.ru, Moscow, Russia), the initial CT combination of nucleotides at markers M5 and M6 was changed for the TC combination using site-directed mutagenesis, and the 928 bp fragments, including the *stc* 5′ regulatory region and 5′ UTR with CT or TC combinations of nucleotides at markers M5 and M6 were cloned into the polylinker of the pGL3-Basic vector (Promega, Madison, WI, USA, https://www.addgene.org/vector-database/2929/ (accessed on 20 August 2026)), which contained cDNA encoding firefly luciferase without any eukaryotic promoter or enhancer sequences. As a result, the *stc* 5′ regulatory sequences were located next to the luciferase gene so that they could regulate its expression. Several plasmids of each type were sequenced, and only plasmids with the correct sequences were used in subsequent experiments.

### 4.7. Transient Transfection and Dual Luciferase Expression Assay

*Drosophila* Schneider S2 cells obtained from the collection of NRC “Kurchatov Institute”—IMG were grown at 25 °C in Schneider’s *Drosophila* Medium (Thermo Fisher Scientific Inc., Waltham, MA, USA) supplemented with 10% heat-inactivated fetal bovine serum (FBS, Thermo Fisher Scientific Inc., Waltham, MA, USA), 50 units/mL penicillin, and 50 µg/mL streptomycin. One milliliter of 2 × 10^6^ cells/mL was seeded per well and rinsed with serum-free medium two hours before transfection. Transient transfection was performed using FuGENE^®^ HD (Roche, Basel, Switzerland) according to the manufacturer’s instructions.

Cells were co-transfected with each of the two vector constructions described above and the pAcRL vector [89] kindly provided by O. G. Maksimenko (1.0 and 0.1 μg per well, respectively). The pAcRL vector expressing Renilla luciferase under the actin promoter was used as a reference to normalize the activity of the experimental reporters.

Transfected cells were analyzed using a Dual-Luciferase Reporter Assay System (Promega, Madison, WI, USA), following the manufacturer’s instructions. Luminescence was measured with a Modulus Microplate Luminometer (Turner BioSystems Inc., Sunnyvale, CA, USA). The relative luciferase activity was determined by calculating the ratio of firefly luciferase luminescence to Renilla luciferase luminescence. The background level of firefly luciferase activity was estimated as the ratio of the luminescence of the pGL3-Basic vector to the luminescence of the pAcRL vector. Four independent plasmid isolations were performed, followed by three cell transformation replicates in each case.

### 4.8. Bioinformatics Assays

The evolutionary conservation of the *stc* 5′ regulatory region was estimated using PhastCons and phastConsElements prediction tracks in the UCSC Genome Browser (https://genome.ucsc.edu/).

Embryonic TFBS hotspots across the entire *stc* 5′ regulatory region were assessed using the modENCODE project analysis [49]. Specific TFBS motifs within the analyzed sequences were predicted using the LASAGNA-search tool [90] with the Transfac 8.3 database and JASPAR 2022 in the UCSC Genome browser with a default score >400, equivalent to *p* = 10 × 10^−4^ (https://genome.ucsc.edu/cgi-bin/hgTracks?db=dm6&hubUrl=http://expdata.cmmt.ubc.ca/JASPAR/UCSC_tracks/hub.txt (accessed on 20 August 2026), [91]) with their respective databases. Additional analysis of TFBS motifs matching SNPs was performed using a 21 nt frame centered on the SNP in question using Tomtom v.5.5.0 with the Combined *Drosophila* Databases at default settings (E < 10, motifs found for both strands) [92].

### 4.9. Statistical Analyses

Parameters characterizing LD, nucleotide diversity (π, θ), and selection (Tajima’s D, Fu and Li’s D* and F*) in the 5′ regulatory region and 5′ UTR of the *stc* gene were calculated using DnaSP 6.12.03 software [93,94]. LD was considered significant if both the Fisher test and the Chi-square test for a pairwise comparison survived Bonferroni correction.

Standard descriptive statistical analysis of lifespan [95,96] was performed to determine the mean lifespan and its accompanying variances, standard deviations, and standard errors; the median, minimum, and maximum lifespans, as well as the lifespans of the lower and upper quartiles and the 10 and 90 percentiles using OASIS 2: Online Application for Survival Analysis 2 [97]. We estimated survival curves using the Kaplan–Meier procedure. SAS Studio v.3.8 University Edition (SAS Institute Inc., Cary, NC, USA) was used to compare frequencies of polymorphic markers (the ANOVA procedure), to analyze the association between polymorphic markers and lifespan, and between polymorphic markers and *stc* transcript amounts (the GLM procedure), to compare lifespans and *stc* transcript amounts in ILs and transfected cells with different polymorphisms (the GLM [means line/Tukey], TTEST and NPAR1WAY [Wilcoxon] procedures), as well as to estimate correlations between mean lifespans and mean *stc* transcript amounts in ILs (the CORR [Spearman] procedure).

## Figures and Tables

**Table 1:** Association between the polymorphic markers and lifespan in the Alexandrov population.

Marker	ANOVA, *p*-Values for Association Between the Marker Allele and Lifespan	Number of ILs	Lifespan (Standard Error of the Mean)	Kruskal–Wallis Test, *p* Values for Differences in Lifespan Between the ILs with Different Marker Alleles
M1 A/G	0.0146	49/45	56.1(0.9)/52.7(1.1)	0.0274
M3 A/T	0.0146	45/48 *	52.7(1.1)/56.1(1.0)	0.0281
M5 C/T	**0.0023**	40/54	56.8(1.1)/52.7(1.0)	0.0067
M6 C/T	**0.0049**	57/37	52.9(1.0)/56.9(1.2)	0.0126
Haplotype GATC/ATCT/ATTC/ATCC/ATTT	0.0466	45/37/8/2/1 ^1^	52.7(1.1) */57.1(1.1) */53.6(2.2)/45.0(4.0)/53.3(7.4)	0.0533

^1^ Data for one IL were excluded. *p* values that survived Bonferroni correction (*p* = 0.05/9 = 0.0056) are in bold. * *p* < 0.05 for multiple comparison of means.

## Data Availability

The original contributions presented in this study are included in the article/Supplementary Materials. Further inquiries can be directed to the corresponding author. The nucleotide sequence data characterized in this study for the Alexandrov population have been deposited in the GenBank database under the accession numbers QB015958–QB016068.

## Supplementary Materials

The following supporting information can be downloaded at: https://www.mdpi.com/article/10.3390/ijms27188343/s1.

## Abbreviations

The following abbreviations are used in this manuscript:

*D. melanogaster*	*Drosophila melanogaster*
IL	Inbred line
LD	Linkage disequilibrium
*stc*	*shuttle craft*
TF	Transcription factor
TFBS	Transcription factor binding site
TSS	Transcription start site

## Appendix A

**Table A1:** Parameters characterizing nucleotide diversity and selection at the 5′ regulatory region and 5′ UTR of the *stc* gene.

Population	Location	Year No. of Sequences	Gene Region (Nucleotide Position)	Number of SNPs	π (s.d.) ^4^	D ^4^
Alexandrov (Russia)	**56.41 N**; 38.72 E; 189 m ^1^	2010 19	**1–868**	**19**	**0.00604 (0.00064)**	**−0.13865**
			1–71	1	0.00741 (0.00056)	1.54705
			72–458	9	0.00734 (0.00094)	0.36185
			459–868	9	0.00455 (0.00067)	−0.95337
		2011 40	**1–868**	**18**	**0.00520 (0.00056)**	**0.21592**
			1–71	1	0.00693 (0.00050)	1.56204
			72–458	10	0.00657 (0.00078)	0.24272
			459–868	7	0.00360 (0.00044)	−0.28395
		2012 18	**1–868**	**15**	**0.00553 (0.00089)**	**0.37841**
			1–71	1	0.00598 (0.00140)	0.86950
			72–458	8	0.00723 (0.00121)	0.70455
			459–868	6	0.00384 (0.00074)	−0.31945
		2014 32	**1–868**	**17**	**0.00518 (0.00056)**	**0.21987**
			1–71	1	0.00656 (0.00079)	1.33679
			72–458	9	0.00677 (0.00077)	0.52933
			459–868	7	0.00344 (0.00049)	−0.55060
Chernobyl (Ukraine)	**51.28 N** 30.21 E 118 m ^1^	2012 33	**1–868**	**22**	**0.00506 (0.00048)**	**−0.65472**
			1–71	1	0.00710 (0.00047)	1.57953
			72–458	10	0.00531 (0.00072)	−0.51261
			459–868	11	0.00447 (0.00048)	−1.02050
Varva (Ukraine)	**50.50 N** 32.72 E 128 m ^1^	2012 28	**1–868**	**19**	**0.00476 (0.00034)**	**−0.54166**
			1–71	3	0.00917 (0.00139)	−0.37405
			72–458	7	0.00472 (0.00045)	0.04440
			459–868	9	0.00403 (0.00045)	−0.90011
Uman (Ukraine)	**48.75 N** 30.22 E 215 m ^1^	2012 35	**1–868**	**21**	**0.00548 (0.00061)**	**−0.22670**
			1–71	2	0.00805 (0.00082)	0.34592
			72–458	12	0.00664 (0.00096)	−0.37513
			459–868	7	0.00395 (0.00046)	−0.13351
Lyon (France) ^2^	**45.77 N** 4.86 E 175 m ^1^	2010 32	**1–868**	**18**	**0.00494 (0.00047)**	**−0.13640**
			1–71	2	0.00894 (0.00116)	0.56027
			72–458	8	0.00508 (0.00066)	−0.02822
			459–868	8	0.00412 (0.00046)	−0.45048
Fiche (Ethiopia) ^3^	**9.81 N** 38.63 E 3070 m ^1^	2011 15	**1–868**	**20**	**0.00778 (0.00100)**	**−0.35358**
			1–71	1	0.00741 (0.00056)	1.54705
			72–458	12	0.00972 (0.00123)	0.07534
			459–868	7	0.00400 (0.00102)	−0.86102
Gikongoro (Rwanda) ^2^	**2.49 S** 28.92 E 1927 m ^1^	2008 25	**1–868**	**23**	**0.00570 (0.00079)**	**−0.81502**
			1–71	3	0.01296 (0.00327)	0.39230
			72–458	14	0.00820 (0.00106)	−0.50021
			459–868	6	0.00208 (0.00071)	−1.38476
Siavonga (Zambia) ^2^	**16.54 S** 28.72 E 530 m ^1^	2010 137	**1–868**	**57**	**0.00874 (0.00021)**	**−0.98729**
			1–71	5	0.01483 (0.00115)	0.31768
			72–458	34	0.01165 (0.00037)	−0.80875
			459–868	18	0.00495 (0.00031)	−1.04531

Nucleotide positions start from the first nucleotide of the sequenced region and are continued according to the reference sequence (conventional sequence coordinates are 2L:15,113,022..15,113,888 using the *D. melanogaster* genome assembly Release 6 plus ISO1 MT (dm6, RefSeq accession: GCF_000001215.4), regardless of indel variation in the natural populations. Nucleotide positions from 1 to 71 correspond to the *CG15269* 3′UTR; nucleotide positions from 72 to 458 correspond to the 5′ regulatory region of *stc*; nucleotide positions from 459 to 868 correspond to the 5′ UTR of *stc*. ^1^ Meters above the sea level. ^2^ http://dpgp.org, [31]. ^3^ http://dpgp.org, [31,32]. ^4^ Parameters were calculated using DnaSP 6.12.03 software [93,94].
